# Paediatric image‐guided radiation therapy: determining and evaluating appropriate kilovoltage planar exposure factors for the Varian on‐board imager

**DOI:** 10.1002/jmrs.352

**Published:** 2019-09-03

**Authors:** John Ryan, David Willis

**Affiliations:** ^1^ School of Health and Biomedical Sciences RMIT University Bundoora Victoria Australia; ^2^ Radiation Therapy Department Sunshine Coast University Hospital Birtinya Queensland Australia

**Keywords:** Exposure, image‐guided radiation therapy, margins, optimisation, paediatrics, phantom imaging, radiation therapy, radiotherapy

## Abstract

**Introduction:**

Kilovoltage (kV) orthogonal imaging is commonly used for image‐guided radiation therapy (IGRT) in paediatrics. Paediatrics have an increased sensitivity to radiation. Exposure factors need to be optimised so that imaging dose is kept as low as reasonably achievable (ALARA).

**Methods:**

A table of low‐dose IGRT radiographic exposure factors for paediatric IGRT was determined through a phantom study. Four anatomical sites, head and neck, thorax, abdomen and pelvis, were investigated. The table was evaluated against standard manufacturer pre‐sets. Dose was evaluated in terms of system‐reported entrance surface air kerma (ESAK). Qualified participants volunteered to perform offline image matching in a further phantom study, recording misalignments detected and providing subjective assessments of image quality using an electronic survey tool. A statistical comparison of matching accuracy was conducted.

**Results:**

Twelve radiation therapists or radiation oncologists completed the image matching task and survey. The low‐dose exposure table reduced imaging dose by 20–94% compared to manufacturer pre‐sets. No significant difference was observed in the accuracy of image matching (head and neck *P* = 0.82, thorax *P* = 0.15, abdomen *P* = 0.33, pelvis *P* = 0.59). Participant image exposure preference was largely equivocal.

**Conclusions:**

Optimising radiographic exposures in paediatric IGRT is feasible, logical and therefore reasonably achievable. Implementation of the low‐dose exposure table presented in this study should be considered by paediatric radiotherapy departments wishing to image gently without compromising the potential to detect set up errors. Further study using a contrast detail phantom and contrast to noise image analysis software is recommended.

## Graphical abstract

Appropriate image dose optimisation for paediatric image‐guided radiation therapy can r esult in a 20–94% reduction in radiation dose, with no decrease in image matching accuracy.

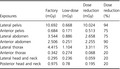

## Introduction

On average, 750 children between 0 and 14 years were annually diagnosed with cancer in Australia between 2010 and 2014.^1^ Radiation therapy (RT) typically plays a role in the management of 25–50% of these cases.^2^, ^3^ Anatomical treatment sites vary depending on the individual malignancy, but commonly treated RT sites include anatomy in the head, thorax and abdomen.^4^ Positively, 5‐year survival rates for paediatric malignancies in Australia have improved from 72% to 84% between the years 1983 and 2013.^1^ This is broadly associated with improved treatment options, a multimodality approach to treatment and international collaboration on clinical trials.^2^

There is a global aim to reduce the radiation exposure for all individuals and especially children undergoing diagnostic examinations. The ‘Image Gently’ campaign is readily recognised and promotes the optimisation of diagnostic exposures in paediatrics.^5^ There have been substantial recent developments in radiology with the establishment of diagnostic reference levels (DRL) in paediatrics,^6^, ^7^ and similar quality improvement initiatives are needed in RT.

Image‐guided radiation therapy (IGRT) is a critical aspect of RT treatment.^8^, ^9^, ^10^ It encompasses a diverse range of imaging technologies, all of which focus on improving the therapeutic ratio between the tumour control probability and normal tissue complication probability.^8^, ^9^ Kilovoltage (kV) on‐board imaging (OBI) is regularly used for IGRT.^10^, ^11^ Modern linear accelerators come equipped with OBI hardware and software. The dose from OBI planar images is low in comparison with clinical RT doses,^12^, ^13^ but may not be as low as reasonably achievable (ALARA).^14^ This is a concern in paediatric patients due to their increased radiosensitivity.^15^, ^16^ Paediatrics have an increased lifetime risk of malignancy of approximately 15% per 1 Gray of X‐rays in comparison with 1–2% per 1 Gray of X‐rays in adults.^15^

Image‐guided radiation therapy helps facilitate a reduction in the negative deterministic effects of RT. Currently, paediatric IGRT is dominated by kV orthogonal imaging and cone beam computed tomography (CBCT)^4^, ^10^ although linac‐based magnetic resonance imaging (MRI) holds great potential.^10^, ^17^ Depending on the specific treatment technique, the frequency of CBCTs during treatment varies.^4^ CBCTs are cautiously scheduled in preference of standard orthogonal radiographic exposures, as although they facilitate soft tissue matching, there is an increased radiation dose to healthy tissues.^12^, ^18^

Kilovoltage CBCTs have a surface dose approximately 10 times higher than planar kV orthogonal imaging.^12^ CBCTs are indicated where soft tissue contrast information is pertinent to the treatment accuracy and bony surrogates or fiducials are insufficient.^10^ There are continuing efforts to move to low‐dose CBCTs; however, paradoxically some low‐dose CBCTs only retain enough contrast for bony anatomy localisation, something readily available with orthogonal kV images.^10^

Due to the deterministic effects of the treatment doses used in RT (e.g. rhabdomyosarcoma 5040 cGy/28#/6 weeks^13^), the focus is to precisely treat only the required anatomy and there is an increased reliance on IGRT. The stochastic effects of radiation associated with imaging of non‐tumour sites for treatment verification are often a secondary concern. However, with the increasing survival statistics, keeping IGRT doses ALARA is paramount.

On‐treatment imaging in RT is used to verify target or target surrogate locations. The OBI exposure factors on a linear accelerator are set by the radiation therapist.^18^ Commonly, pre‐sets are selected for consistency and workflow. The number of pre‐sets available is limited and usually defined by the manufacturer for adult patients. On the *Varian Clinac^®^ iX* (Varian Medical Systems, Palo Alto, CA) for instance, there is a single pre‐set of exposure factors for anterior–posterior abdomen and another for lateral abdomen. There is no differentiation for separation, such as large, medium and small. Standard practice would include taking a lateral and anterior or posterior image projection pair. A posterior image is recommended when imaging the head to limit lens dose.^19^ Appropriate imaging exposure setting pre‐sets avoids unnecessary imaging dose to the patient, repeat imaging and extended treatment time.

Optimisation of paediatric exposures for diagnostic examinations is frequently undertaken.^16^, ^20^, ^21^ Paediatric phantoms are used to simulate the clinical environment.^20^ The radiographic exposure setting can be adjusted, and the contrast‐to‐noise ratio or diagnostic accuracy measured. In RT, the validity of the exposure setting can be measured by how accurately the radiation therapist matches acquired images when they are blinded to the known offsets and image exposure settings. It is important that the order, in which the therapist matches the images acquired with the different exposure factors, is varied to prevent any biases such as matching fatigue.

The *International Commission on Radiation Units and Measurements* (ICRU) Report 74^22^ outlines that entrance surface air kerma (ESAK; automatically calculated on the *Varian Clinac^®^ iX*) as an appropriate measure to compare radiation outputs and, thus, a method to compare exposure settings.

Optimising imaging exposure in RT has focused on the changing modalities available, that is megavoltage (MV), kV and CBCT.^19^ Although it is common to optimise specific types of exposures in radiology, this is only really happening for CBCT in RT and refinement of kV orthogonal imaging settings is not widely published. There has been a sustained international effort to establish DRLs in radiology for paediatrics, but no similar guidelines exist for IGRT.

### Aim

To define a set of exposure factors for paediatric IGRT on a *Varian* OBI system that reduces imaging dose while maintaining clinical utility.

### Research question

Can the accuracy of IGRT for paediatric patients be maintained using reduced radiographic dose exposures instead of vendor pre‐sets?

## Methods and Materials

Hospital ethical permission (*Application* AU201512‐10) was granted prior to commencing the study. The research team had access to a *Philips Big Bore* ^®^ Computer Tomography (CT) Scanner (Philips Healthcare, Amsterdam, the Netherlands), *Varian Eclipse* ^®^ Version 15, *Varian ARIA* ^®^ Oncology Information System, *Varian Clinac^®^ iX* Linear Accelerator (Varian Medical Systems), a CIRS ATOM^®^ Model 705 anthropomorphic phantom (CIRS, Norfolk, VA) representing a 5‐year‐old child (affectionately known as ‘Mikey’), a shared patient database with a 2nd RT department and radiation therapists and radiation oncologists across both sites. ESAK was used to compare the radiation dose delivered to a paediatric anthropomorphic phantom. No dosimetric measurements on actual patients were taken. All software and hardware used in this project were in clinical use and underwent regular quality assurance checks.

### Deriving the low‐dose exposure factors

**Figure 1:**
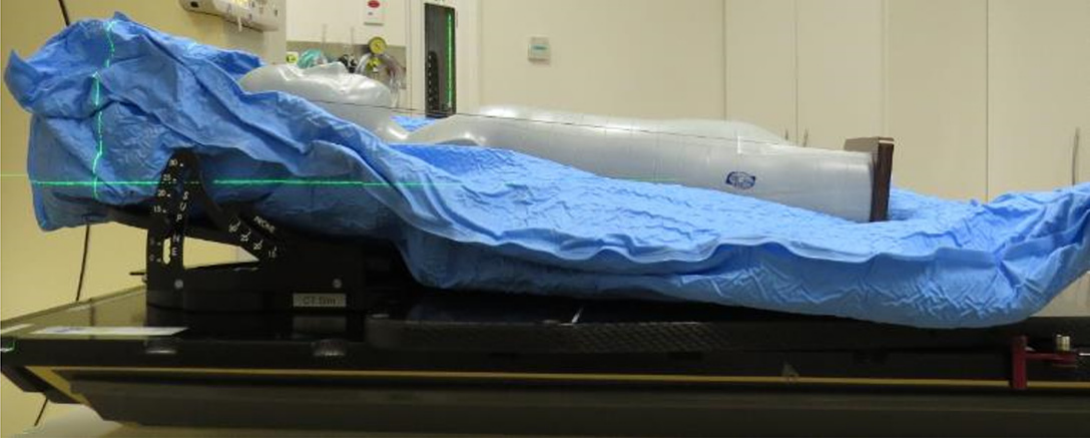
CIRS ATOM ® Model 705 anthropomorphic phantom in scanning position.

**Figure 2:**
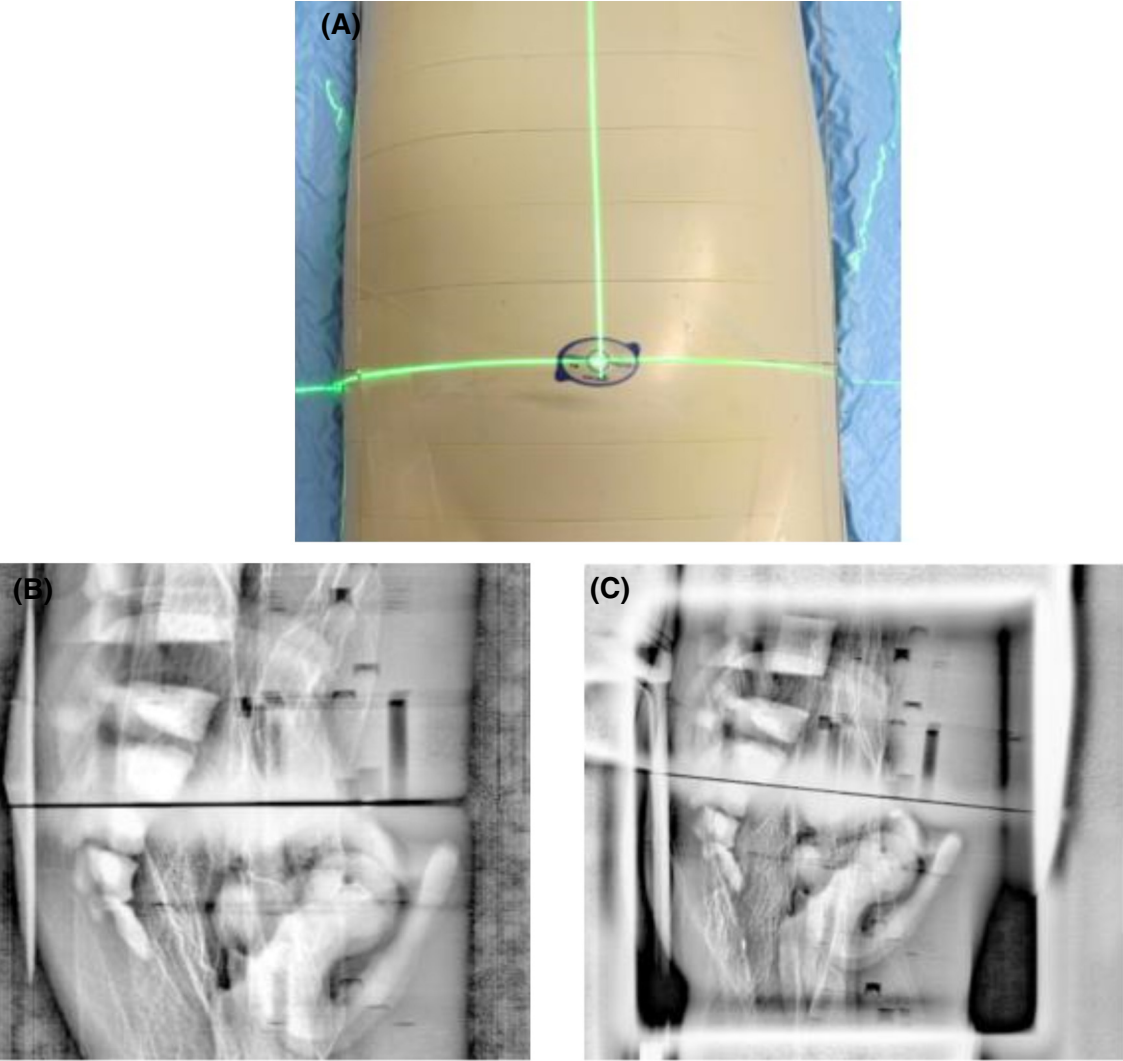
(A) Slab phantom, (B) transmission artefact with no couch rotation, (C) reduced transmission artefact when 10‐degree couch rotation.

- A test patient for the CIRS ATOM^®^ Model 705 anthropomorphic phantom, ‘Mikey’, was created in the RT electronic shared database.
- The anthropomorphic phantom (Mikey) was set up in a standard and reproducible fashion (Fig. 1), and a CT scan was acquired. Mikey was scanned on a 15‐degree incline and planned with a 10‐degree couch angle. This was done to avoid direct X‐ray transmission artefacts through the individual phantom slabs (Fig. 2).
- Four standard paediatric RT sites, head and neck, thorax, abdomen and pelvis, were selected for comparison. The control arm was the factory installed pre‐set exposure factors on the *Varian Clinac^®^ iX* Linear Accelerator. Where more than one factory pre‐set existed for an anatomical site, the option with the lowest milliamperage exposure length (mAs) was selected.
- Four radiotherapy plans with orthogonal set up imaging fields and associated digital reconstructed radiographs (DRRs) were created. Anterior and right lateral orthogonal image sets were created for the pelvis, abdomen, and thorax. A posterior and right lateral orthogonal image set was created for the head and neck.
- These plans were scheduled on the Varian Clinac^®^ iX Linear Accelerator and images acquired with the factory pre‐set exposure factors, and subsequent variation of the milliamperage, exposure length and focal spot size are proportional to exposure dose. The focal spot size will affect image acuity with a smaller focal spot size having reduced image penumbra.
- mAs was systematically reduced until the acquired images were noticeably degraded. This was defined as the cut‐off point. The exposure setting chosen for the low‐dose pre‐sets was one level higher (approximately 25% greater dose) than the defined cut‐off, to account for patient size variability and reduce the potential need to reimage due to unsatisfactory image quality. Two experienced radiation therapists (5+ years post‐qualification) ultimately decided on a low‐dose pre‐set exposure table (Table 1).
- All images were reviewed using the *Varian* software content filter. The source axis distance (SAD) was 100 cm, and image detector positions were vertical 50 cm, long 0 cm and lateral 0 cm for all images.
- Image exposure settings and associated ESAK dose were recorded and analysed in *Microsoft^®^ Excel* version *Microsoft^®^ Office 365* (Table 2).

**Table 1:** Image exposure table.

Site and projection	Exposure table	Focal spot size	kV	mA	ms	mAs	Dose mGy
Pelvis							
Lateral	Factory	Large	105	200	400	80	10.692
	Low‐dose	Small	105	125	40	5	0.668
Anterior	Factory	Large	75	200	50	10	0.684
	Low‐dose	Small	75	50	50	2.5	0.171
Abdomen							
Lateral	Factory	Large	85	200	200	40	3.544
	Low‐dose	Small	85	125	80	10	0.886
Anterior	Factory	Large	80	200	160	32	2.506
	Low‐dose	Small	80	200	16	3.2	0.251
Thorax							
Lateral	Factory	Large	95	200	200	40	4.415
	Low‐dose	Small	95	125	80	10	1.104
Anterior	Factory	Large	75	200	25	5	0.342
	Low‐dose	Small	75	200	20	4	0.274
Head and neck							
Lateral	Factory	Large	70	200	25	5	0.295
	Low‐dose	Small	70	200	20	4	0.236
Posterior	Factory	Large	100	200	40	8	0.975
	Low‐dose	Small	100	160	40	6.4	0.78

**Table 2:** Dose comparison between factory and low‐dose pre‐set exposure recommendations.

Exposures	Factory (mGy)	Low‐dose (mGy)	Dose reduction (mGy)	Dose reduction (%)
Lateral pelvis	10.692	0.668	10.024	94
Anterior pelvis	0.684	0.171	0.513	75
Lateral abdomen	3.544	0.886	2.658	75
Anterior abdomen	2.506	0.251	2.255	90
Lateral thorax	4.415	1.104	3.311	75
Anterior thorax	0.342	0.274	0.068	20
Lateral head and neck	0.295	0.236	0.059	20
Posterior head and neck	0.975	0.78	0.195	20

### Evaluation of the clinical utility of the derived table of exposure factors

For purposes of blinding the participants and removing potential sources of error, a series of set up ‘misalignments’ were created digitally as follows:

- A routine plan was created for each of the four anatomical sites being investigated using *Eclipse^®^ Version 15*. Each plan had orthogonal imaging available. Each plan was copied twice. In each of the copied plans, the isocentre was altered by a pre‐planned distance in each of the three cardinal directions (Table 3).
- Mikey was set up on the *Varian Clinac^®^ iX* as per his CT scan for each area. The first plan on each site was used for set up verification. After confirming that Mikey was positioned correctly, the subsequent two plans were called up and imaged without moving Mikey, with one plan employing the factory pre‐set and the other plan using low‐dose pre‐set exposure factors. This resulted in verification images with known offsets for each set of exposure factors. The order of exposure for plans 2 and 3 was random. Images on each site were archived in the *Varian ARIA* ^®^ software application offline review.
- A *SurveyMonkey* 23 survey was created with a participant information form and a guide on how to participate in the image matching exercises using offline review (Supporting information I and II).
- All (*n* = 62) current radiation therapists and radiation oncologists with access to the image database were emailed with an invitation to participate and a study explanatory statement.
- Participation was voluntary, and all participants were blinded to the known offsets. Participants were asked to perform offline image matching in a manner consistent with clinical practice. Detected offsets and subjective assessments of image quality were recorded using *SurveyMonkey*. The survey was open for a set period of 2 months. All participants' identifiable details were anonymised using *SurveyMonkey*.
- Participant image matching data from *SurveyMonkey* were exported to* Microsoft^®^ Excel* version *Microsoft^®^ Office 365*.
- Obvious transcription errors (opposite direction, magnitude >0.4 cm), where the recorded moves had an incorrect sign due to participants transcribing moves from *Offline Review* to *SurveyMonkey*, were rectified (e.g. planned offset 0.4 cm, recorded move −0.4cm).
- The sum of the scalar discrepancies between known image offsets and detected offsets recorded in *SurveyMonkey* was calculated for each matched orthogonal image pair (Supporting information III).
- Five paired sample t‐tests were carried out using *IBM SPSS Statistics 2* 24 to compare the mean scalar discrepancies between the participants for the four anatomical sites reviewed and all the anatomical sites collectively with both sets of exposures (Supporting information IV).
- Individual participant performance was assessed to see whether matching accuracy varied depending on the exposure setting.

**Table 3:** Planned image offsets.

Plan name	Vertical offset (cm)	Longitudinal offset (cm)	Lateral offset (cm)	Exposure setting used
Pelvis	0	0	0	Factory pre‐sets
Pelvis A	0.3 Ant	0.7 Inf	0.5 RT	Factory pre‐sets
Pelvis B	0.6 Ant	0.4 Sup	0.2 LT	Low‐dose pre‐sets
Abdomen	0	0	0	Factory pre‐sets
Abdomen C	0.6 Ant	0.7 Inf	0.4 RT	Low‐dose pre‐sets
Abdomen D	0.9 Ant	0.4 sup	0	Factory pre‐sets
Thorax	0	0	0	Factory pre‐sets
Thorax E	1.0 post	0.5 Inf	0.6 LT	Low‐dose pre‐sets
Thorax F	0.2 post	0.1 sup	0.7 RT	Factory pre‐sets
Head and neck	0	0	0	Factory Pre‐sets
Head and neck G	0.4 ant	0.3 inf	0.7 RT	Factory pre‐sets
Head and neck H	0.0	0.8 sup	0.6 LT	Low‐dose pre‐sets

## Results

**Figure 3:**
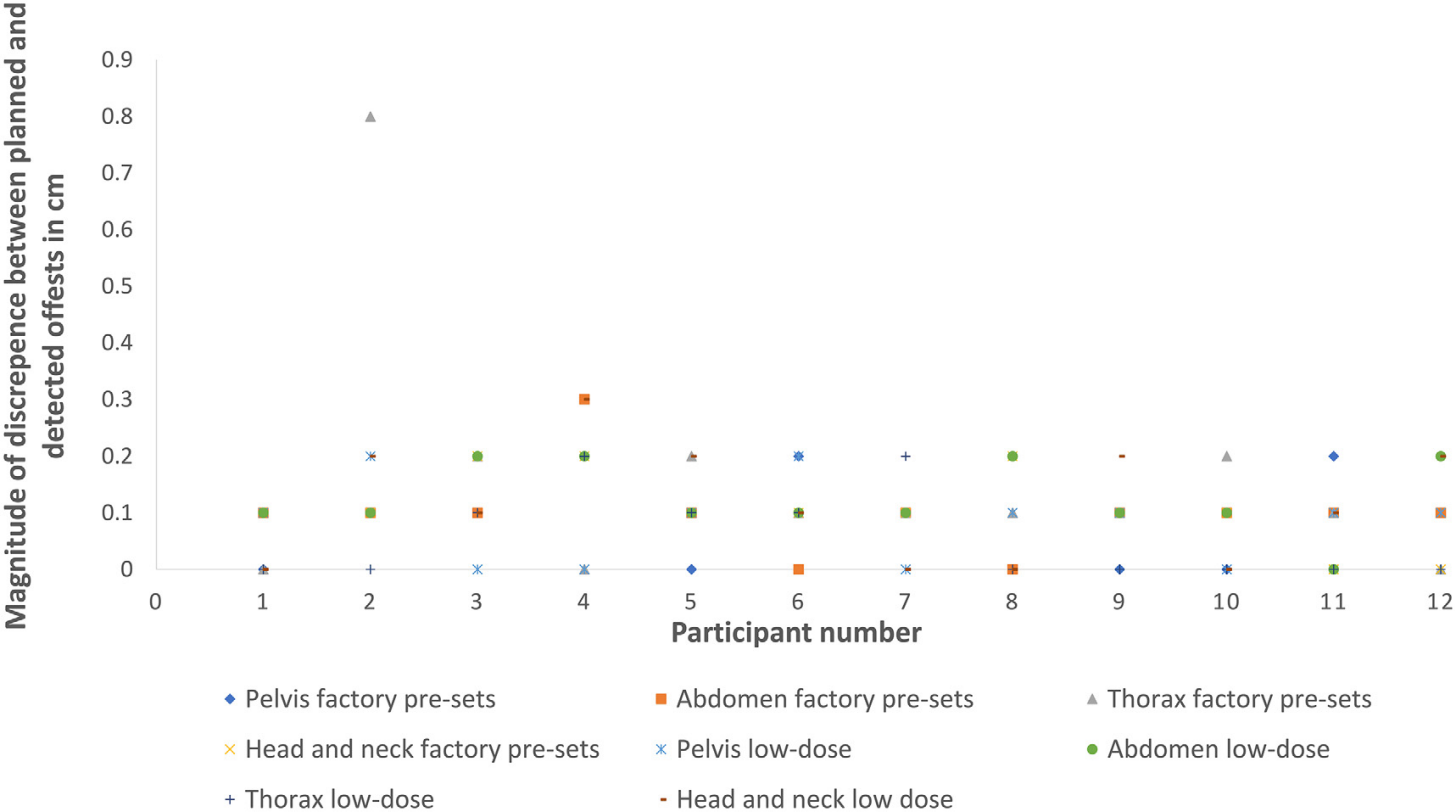
Overview of discrepancies between recorded and planned moves.

**Figure 4:**
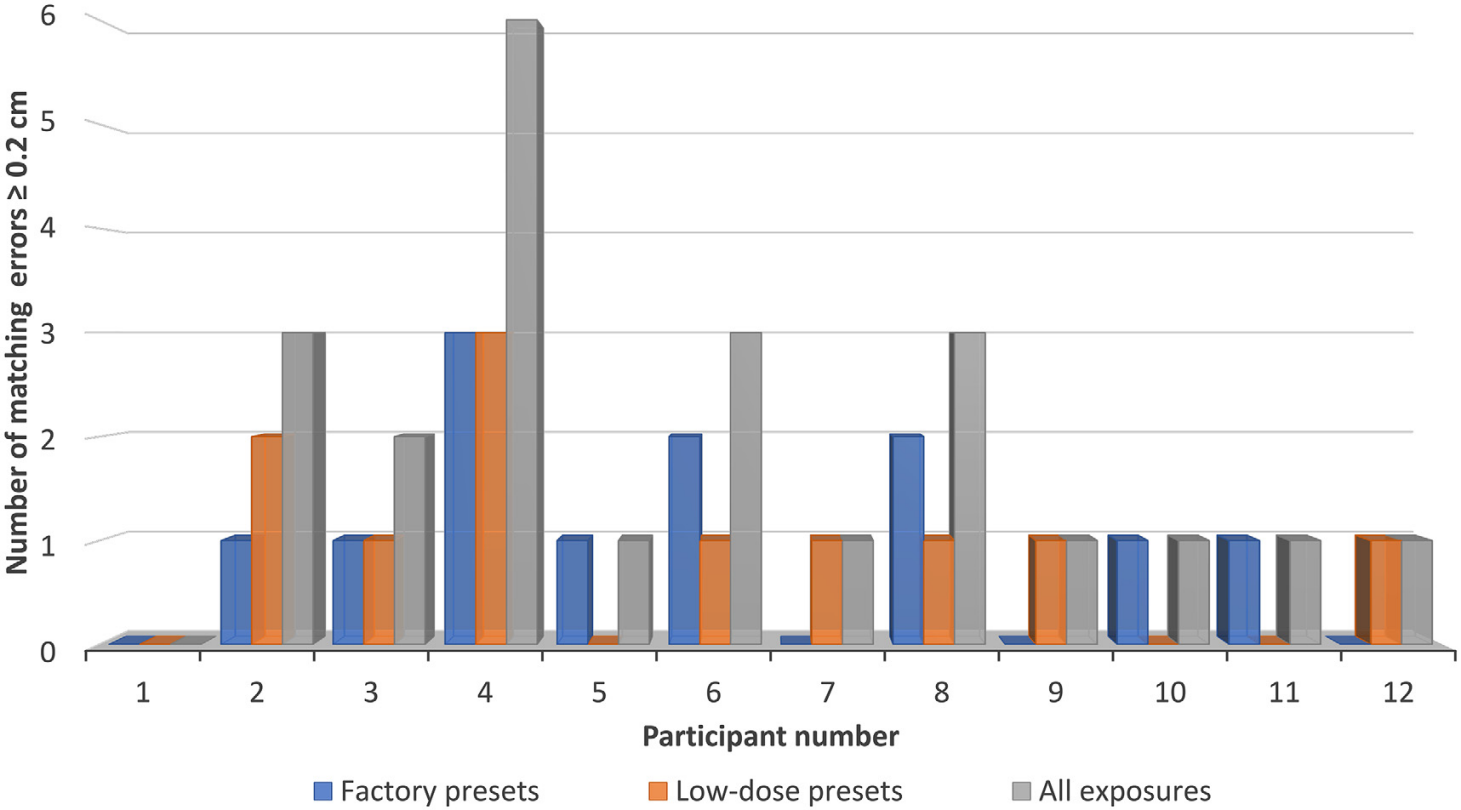
Number of orthogonal image pairs with errors in offset detection of ≥0.2 cm per participant.

**Figure 5:**
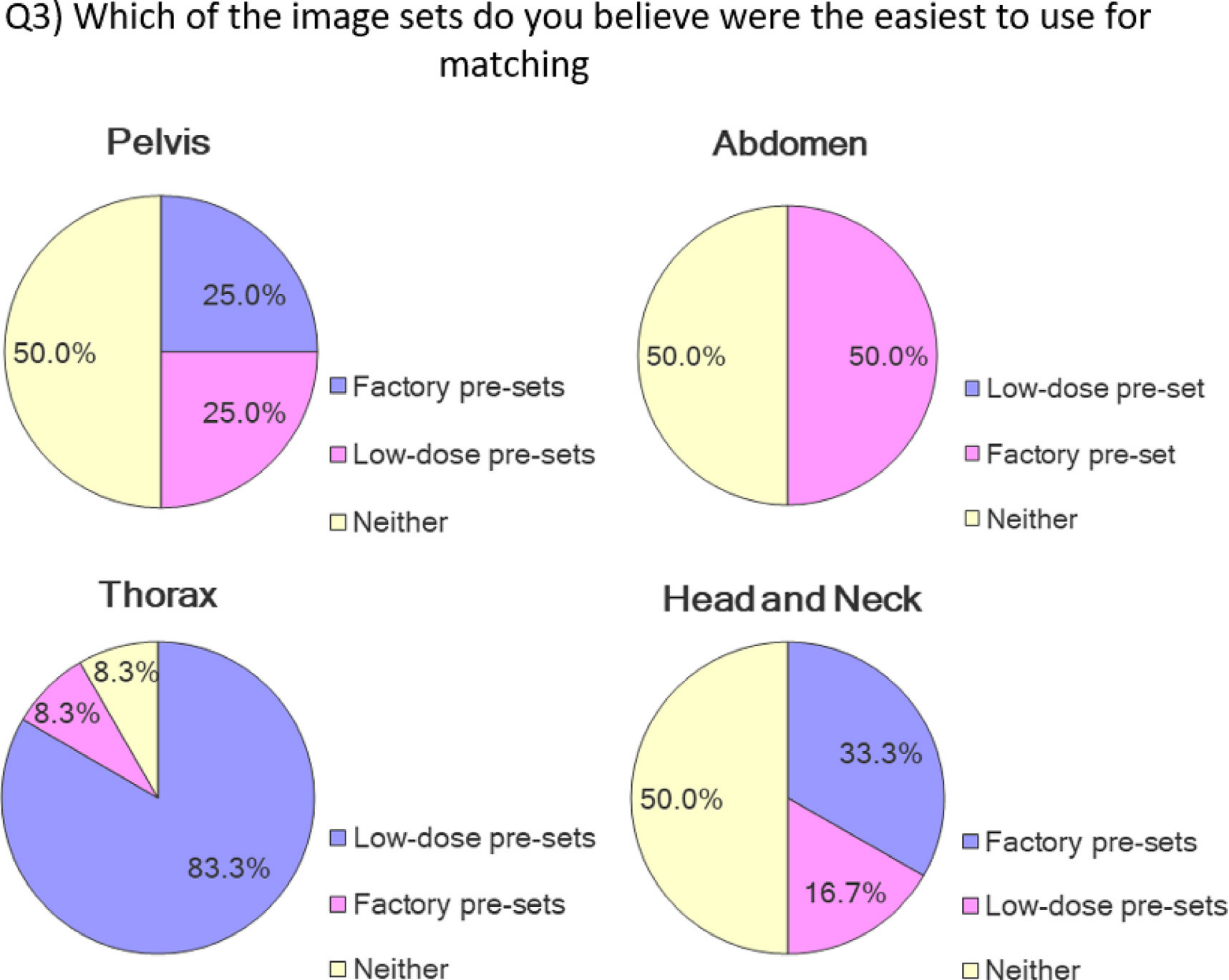
Participant exposure preference for IGRT.

A dose comparison of the factory pre‐sets and the low‐dose pre‐set exposure recommendations is presented in Table 2. Twelve of the sixty‐two emailed radiation therapists or radiation oncologists completed the image matching surveys (19.35%). An overview of the magnitude of the discrepancies between the planned moves and the recorded offsets is presented in Figure 3. There were 96 matched image pairs. Ninety‐three (96.9 %) and 71 (73.9%) of the matched image pairs had a summed scalar matching error of ≤0.2 and ≤0.1 cm, respectively. The discrepancies between planned and recorded moves for participant 2 when using the factory pre‐sets to match in the thorax region were the largest in the cohort. However, no statistical difference was identified in the study sample or in the ability of trained staff to accurately identify misalignment with either set of exposure factors (Table 4). Participants were equally likely to have a summed scalar matching error ≥0.2 cm with either the factory pre‐sets or the low‐dose pre‐sets (Fig. 4). In total, 23 (23.9%) orthogonal image pairs had matching errors ≥0.2 cm, 11 (11.4%) with the low‐dose pre‐sets and 12 (12.5%) with the factory pre‐sets. Qualitative information on participant image set preference is presented in Figure 5.

**Table 4:** Mean differences between matched and planned moves.

Exposure setting	Image set in *SurveyMonkey*	Mean scalar difference between detected offsets and planned offsets (cm)	Paired sample t‐test	
			*T* value	*P* value (2‐tailed)
Pelvis factory pre‐sets	A	0.100	0.561	0.586
Pelvis low‐dose pre‐sets	B	0.080		
Abdomen factory pre‐set	C	0.100	−1.000	0.339
Abdomen low‐dose pre‐sets	D	0.125		
Thorax factory pre‐sets	F	0.166	1.545	0.151
Thorax low‐dose pre‐sets	E	0.058		
Head and neck factory pre‐sets	G	0.108	−0.233	0.820
Head and neck low‐dose pre‐sets	H	0.116		
All anatomical sites factory pre‐sets	N/A	0.118	1.017	0.314
All anatomical sites low‐dose pre‐sets	N/A	0.095		

## Discussion

The exposure factors defined and, subsequently, tested had substantial reductions in mAs compared to factory pre‐sets. The corresponding ESAK imaging doses predicted by the system were reduced for all studied sites and projections, ranging from 20% to 94%. The largest dose reductions (≥75%) were for the pelvis, abdomen, and lateral thorax exposures. Further reductions were possible while maintaining a usable image of the phantom; however, these lower settings were not recommended for clinical use due to concerns about the verisimilitude of the phantom and the potential for a clinical under‐exposure. If such an image had insufficient clinical utility, a second exposure would be required which is contrary to the objective of minimising imaging dose.

The subsequent testing demonstrated equivalence between the two sets of exposure factors in terms of the ability of clinical staff to accurately detect misalignment. The method of creating these misalignments (or offsets) eliminated several sources of error, for example, errors in positioning of the treatment table if manual adjustments had been used. This method along with the approach of using offline review tools also blinded participants to the offsets.

The *Varian Clinac^®^ iX* linear accelerator ESAK dose readout does not consider field size. Field size was set for the anatomical site under review, and the settings were kept consistent between the different dose exposures for each site. ESAK is a measure of machine output and valid to compare exposures but does not give the exact dose delivered to the patient or phantom per exposure.

The paired use of the *SurveyMonkey* questionnaire and offline review was novel and enabled a larger number of participants to verify the appropriateness of the low‐dose pre‐set exposure table than otherwise possible. As well as making efficient use of linear accelerator time, it reduced the logistical challenges of keeping participants blinded to the exposure recommendations, as participants did not have to be present when the images were acquired.

The percentage imaging dose reduction in Table 2 could be clinically significant given the frequency of imaging for paediatric IGRT. The image dose reduction varied between 0.059 and 10.024 mGy depending on the site and image projection. For example, a paediatric patient receiving pelvic treatment for a rhabdomyosarcoma (5040 cGy/28#/6 weeks^13^), imaged daily using the factory or low‐dose pre‐sets would receive 318.5 or 23.5 mGy, respectively.

Participant preference between the low‐dose pre‐set and factory pre‐set images varied depending on the anatomical site and was ambiguous. Figure 5 presents these qualitative findings. The difference in the matched moves for individual participants is clinically negligible (≤0.1 cm). Several participants commented on the poor quality of the pre‐set exposure for the lateral chest image, though no statistically significant difference in matching accuracy was observed. Interestingly, the only likely clinically significant image matching error recorded was demonstrated by participant 2 (scalar magnitude of error 0.8 cm) using the factory pre‐sets and image matching in the thorax. This may be an outlier or possible transcription error. In a clinical scenario, it is unlikely that a therapist or oncologist would match using the lateral thorax factory pre‐sets as the resultant image is overexposed. Survey responses on other imaging sites and projections did not highlight any clear user preference for either set of images.

There were some limitations and recommendations with this study. Visually deciding on an appropriate low‐dose exposure table was challenging and subjective. Substantial time (approximately 30 hours) was given to acquiring and comparing images. This study was focused on the application of the low‐dose pre‐sets in line with the clinical environment. The assessment was thus kept similar to the clinical tasks. Assessing the exposure recommendations using a contrast detail phantom and contrast to noise detection software is recommended for future studies.

As the survey participants were anonymous, there was no way of knowing who completed the image matching tasks and their relative experience level. Transcription errors were anticipated, particularly with values being recorded as positive or negative. Future studies may consider limiting offsets to positive values only or recording matched moves in offline review. The methodology employed could be used when testing the clinical utility of different IGRT imaging technology, such as MRI versus CBCT, and comparing imaging protocols.

## Conclusion

The exposure table defined in this study reduced imaging dose without compromising the accuracy of misalignment detection. Utilising more patient‐specific exposure factors is logical, readily achievable and consistent with the ALARA principle. The low‐dose pre‐set table should be considered for use within paediatric radiotherapy departments.

## Conflict of Interest

The authors declare no conflict of interest.
