# Supplementary material for: Paediatric image‐guided radiation therapy: determining and evaluating appropriate kilovoltage planar exposure factors for the Varian on‐board imager
Source: J Med Radiat Sci. 2019 Sep 3;67(1):16–24. doi: 10.1002/jmrs.352 (PMC7063249; doi:10.1002/jmrs.352)
Supplement: Supplementary file 1 [file JMRS-67-16-s001.docx]

Supporting Information

| I) Invitation to participate email | Participant information email |
| --- | --- |
| II) *Survey Monkey* Questionnaire | Example of questionnaires completed by participants |
| III) Image matching data | All participant image matching data |
| IV) Statistical analyses | Results from the statistical analyses tests completed |

I) Invitation to participate email

“Introduction:

We are seeking Radiation Therapists and Oncologists and to voluntarily assist with a quality improvement project.

Purpose:

We have produced a table of recommended OBI exposure factors for imaging small children that aim to reduce dose while maintaining effectiveness. This part of the project will compare the new factors with *Factory*’s pre-set OBI exposure factors in terms of accurate detection of setup variations.

Procedures:

If you agree to participate, we will ask you to perform offline 2D-2D image matching on the Mikey phantom (anthropomorphic phantom of a 5-year-old) in 4 regions of the body (head, thorax, abdomen, pelvis).

You will be asked to match on two image sets for each of the 4 regions of the body and document the recorded moves in this online survey. Each of the image sets were acquired using know exposures and have different offsets. It is recommended to use the *Varian* software content filter when reviewing images.

We will also ask your opinion on the relative quality of the images and ease of use for matching purposes.

As we will not be timing the image matching processes, we request that you proceed at a pace consistent with clinical practice. It is not essential that all images are matched in a single session. We estimate that it will take around 20-30 minutes in total to complete this activity.

Risks and Benefits:

This activity will not be used as an assessment of your proficiency, i.e. it is not a performance appraisal or a competition. The results of the matching accuracy will only be presented as grouped data in any resulting publications or presentations. This means your individual matching outcomes will not be identifiable.

Participation is voluntary:

Your decision to participate, not participate or cease participation will not affect your relationship with the project team or North West Cancer Centre. Thank you in advance.

Link to Survey: <https://www.surveymonkey.com/r/BeamKids>”

II) *Survey Monkey* Questionnaire


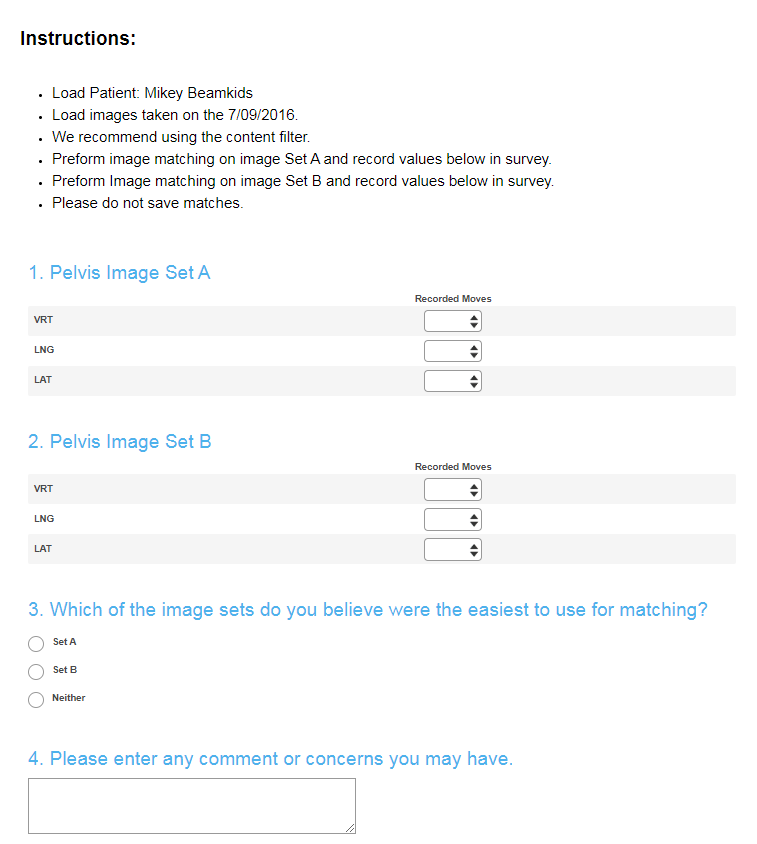


III) Image matching data

Pelvis


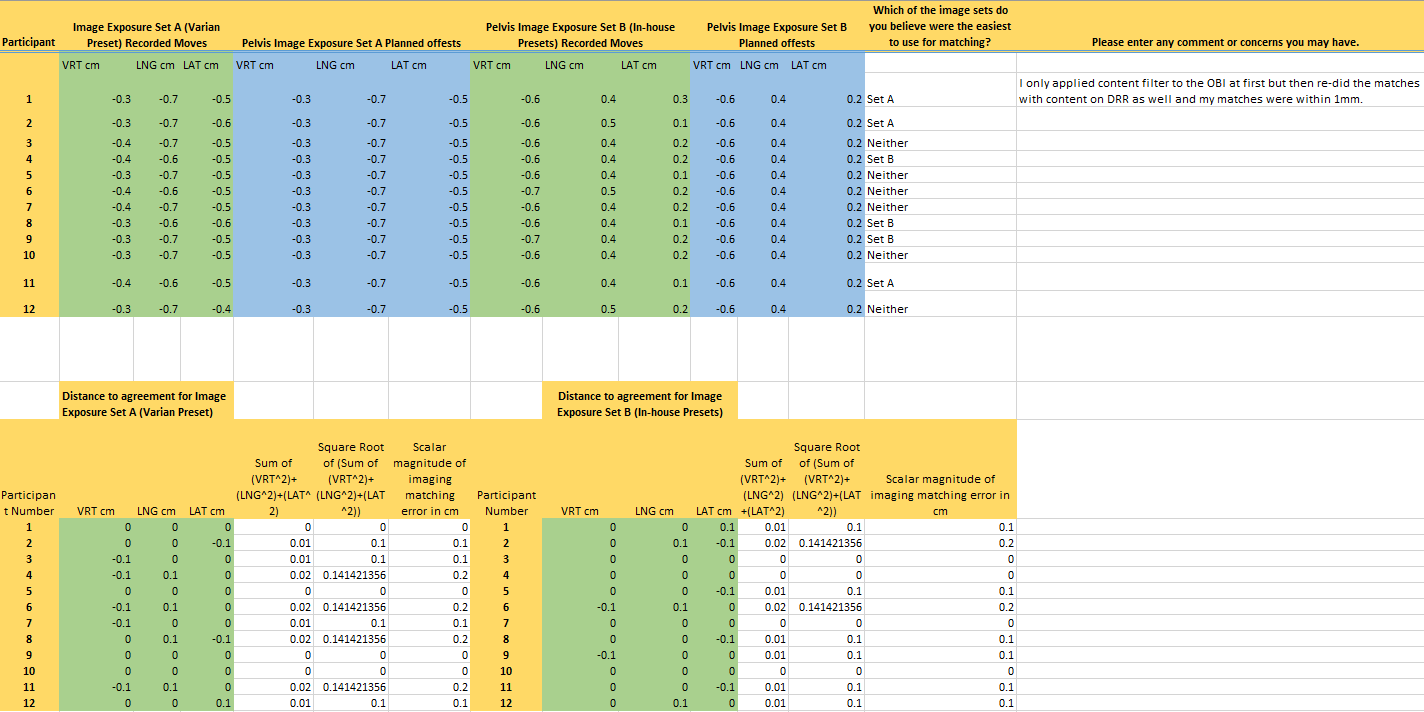


B) Abdomen


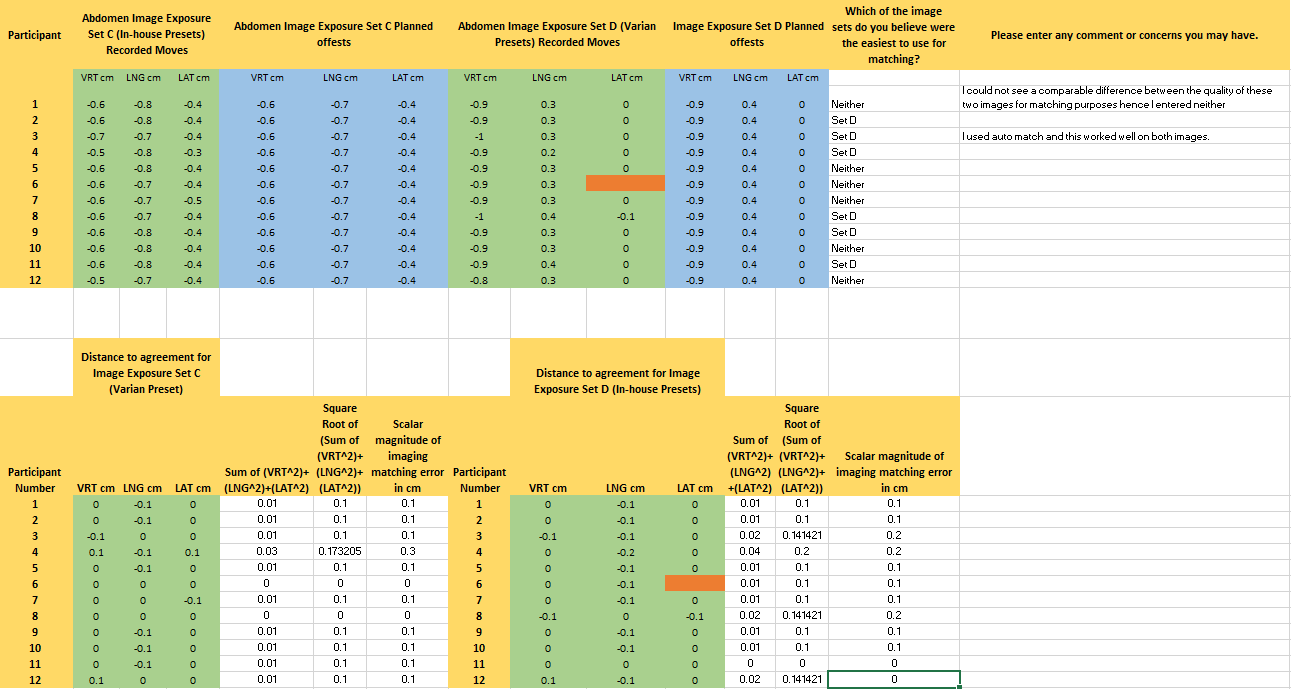


C) Thorax


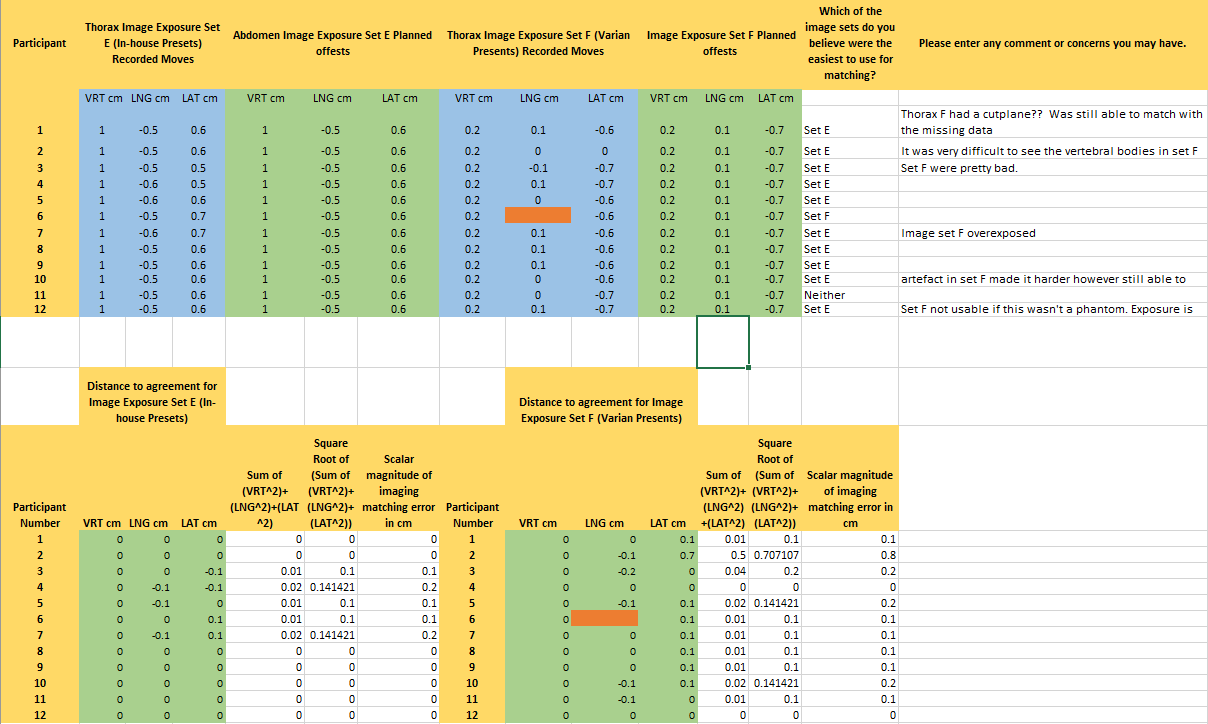


D) Head and Neck


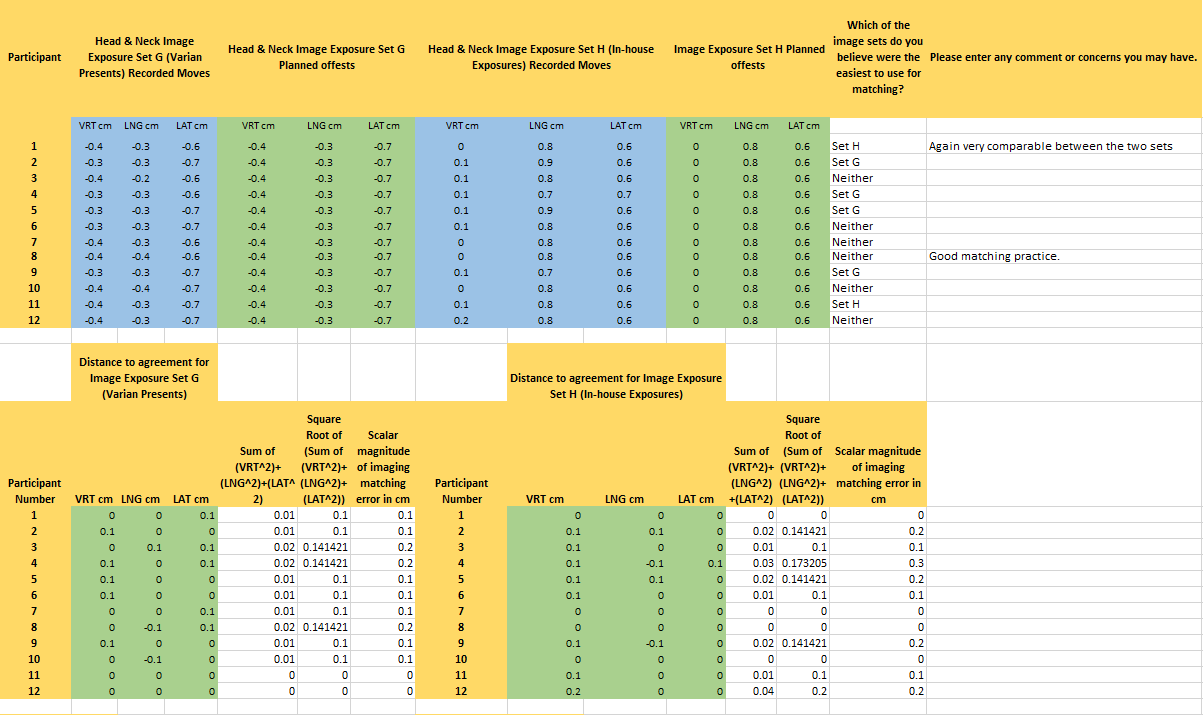


E) Participant number of offsets ≥ 0.2 cm


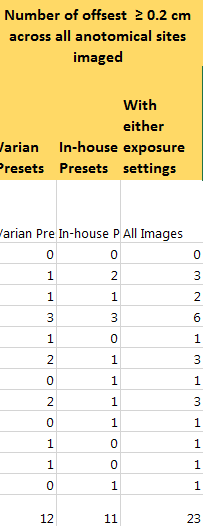


IV) Statistical analyses

Paired Sample T Test Statistics

| **Paired Samples Statistics** | | | | | |
| --- | --- | --- | --- | --- | --- |
|  | | Mean | N | Std. Deviation | Std. Error Mean |
| Pair 1 | Scalar magnitude of image matching error for the Pelvis anatomical site using Varian imaging presets | .1000000 | 12 | .08528029 | .02461830 |
|  | Scalar magnitude of image matching error for the Pelvis anatomical site using In-house imaging presets | .0833333 | 12 | .07177406 | .02071939 |
| Pair 2 | Scalar magnitude of image matching error for the Abdominal anatomical site using Varian imaging presets | .1000000 | 12 | .07385489 | .02132007 |
|  | Scalar magnitude of image matching error for the Abdominal anatomical site using In-house imaging presets | .1250000 | 12 | .06215816 | .01794351 |
| Pair 3 | Scalar magnitude of image matching error for the Chest anatomical site using Varian imaging presets | .1666667 | 12 | .21033883 | .06071959 |
|  | Scalar magnitude of image matching error for the Chest anatomical site using In-house imaging presets | .0583333 | 12 | .07929615 | .02289083 |
| Pair 4 | Scalar magnitude of image matching error for the Head and Neck anatomical site using Varian imaging presets | .1083333 | 12 | .06685579 | .01929960 |
|  | Scalar magnitude of image matching error for the Head and Neck anatomical site using In-house imaging presets | .1166667 | 12 | .10298573 | .02972942 |
| Pair 5 | Scalar magnitude of image matching error for the All anatomical sites using Varian imaging presets | .1187500 | 48 | .12317821 | .01777924 |
|  | Scalar magnitude of image matching error for the All anatomical sites using In-house imaging presets | .0958333 | 48 | .08240619 | .01189431 |

| **Paired Samples Correlations** | | | | |
| --- | --- | --- | --- | --- |
|  | | N | Correlation | Sig. |
| Pair 1 | Scalar magnitude of image matching error for the Pelvis anatomical site using Varian imaging presets & Scalar magnitude of image matching error for the Pelvis anatomical site using In-house imaging presets | 12 | .149 | .645 |
| Pair 2 | Scalar magnitude of image matching error for the Abdominal anatomical site using Varian imaging presets & Scalar magnitude of image matching error for the Abdominal anatomical site using In-house imaging presets | 12 | .198 | .537 |
| Pair 3 | Scalar magnitude of image matching error for the Chest anatomical site using Varian imaging presets & Scalar magnitude of image matching error for the Chest anatomical site using In-house imaging presets | 12 | -.254 | .425 |
| Pair 4 | Scalar magnitude of image matching error for the Head and Neck anatomical site using Varian imaging presets & Scalar magnitude of image matching error for the Head and Neck anatomical site using In-house imaging presets | 12 | -.022 | .946 |
| Pair 5 | Scalar magnitude of image matching error for the All anatomical sites using Varian imaging presets & Scalar magnitude of image matching error for the All anatomical sites using In-house imaging presets | 48 | -.118 | .425 |

| **Paired Samples Test** | | | | | | | | | |
| --- | --- | --- | --- | --- | --- | --- | --- | --- | --- |
|  | | Paired Differences | | | | | t | df | Sig. (2-tailed) |
|  |  | Mean | Std. Deviation | Std. Error Mean | 95% Confidence Interval of the Difference | |  |  |  |
|  |  |  |  |  | Lower | Upper |  |  |  |
| Pair 1 | Scalar magnitude of image matching error for the Pelvis anatomical site using Varian imaging presets - Scalar magnitude of image matching error for the Pelvis anatomical site using In-house imaging presets | .01666667 | .10298573 | .02972942 | -.04876734 | .08210068 | .561 | 11 | .586 |
| Pair 2 | Scalar magnitude of image matching error for the Abdominal anatomical site using Varian imaging presets - Scalar magnitude of image matching error for the Abdominal anatomical site using In-house imaging presets | -.02500000 | .08660254 | .02500000 | -.08002463 | .03002463 | -1.000 | 11 | .339 |
| Pair 3 | Scalar magnitude of image matching error for the Chest anatomical site using Varian imaging presets - Scalar magnitude of image matching error for the Chest anatomical site using In-house imaging presets | .10833333 | .24293034 | .07012795 | -.04601724 | .26268391 | 1.545 | 11 | .151 |
| Pair 4 | Scalar magnitude of image matching error for the Head and Neck anatomical site using Varian imaging presets - Scalar magnitude of image matching error for the Head and Neck anatomical site using In-house imaging presets | -.00833333 | .12401124 | .03579896 | -.08712632 | .07045965 | -.233 | 11 | .820 |
| Pair 5 | Scalar magnitude of image matching error for the All anatomical sites using Varian imaging presets - Scalar magnitude of image matching error for the All anatomical sites using In-house imaging presets | .02291667 | .15606816 | .02252650 | -.02240080 | .06823414 | 1.017 | 47 | .314 |
